# Impairments of the primary afferent nerves in a rat model of diabetic visceral hyposensitivity

**DOI:** 10.1186/s12990-015-0075-5

**Published:** 2015-12-10

**Authors:** Li Dong, Xizi Liang, Biying Sun, Xiaowei Ding, Hongxiu Han, Weifang Rong, Guohua Zhang

**Affiliations:** Hongqiao International Institute of Medicine, Shanghai Tongren Hospital, Shanghai Jiaotong University School of Medicine, 1111 Xianxia Road, Shanghai, 200050 China; Department of Physiology, Faculty of Basic Medicine, Shanghai Jiaotong University School of Medicine, 280 South Chongqing Road, Shanghai, 200025 China; Department of Pathology, Shanghai Ninth People’s Hospital, Shanghai Jiaotong University School of Medicine, 659 Zhizhaoju Road, Shanghai, 200011 China

**Keywords:** Diabetes, Visceral hyposensitivity, Primary afferents, Calcitonin gene-related peptide (CGRP), Protein gene product 9.5 (PGP 9.5)

## Abstract

**Background:**

Diabetic neuropathy in visceral organs such as the gastrointestinal (GI) tract is still poorly understood, despite that GI symptoms are among the most common diabetic complications. The present study was designed to explore the changes in visceral sensitivity and the underlying functional and morphological deficits of the sensory nerves in short-term diabetic rats. Here, we compared the colorectal distension (CRD)-induced visceromotor response (VMR, an index of visceral pain) in vivo, the mechanosensitivity of colonic afferents ex vivo as well as the expression of protein gene product (PGP) 9.5 and calcitonin gene-related peptide (CGRP) in colon between diabetic (3–6 weeks after streptozotocin injection) and control (age-matched vehicle injection) rats.

**Results:**

VMR was markedly decreased in the diabetic compared to the control rats. There was a significant decrease in multiunit pelvic afferent nerve responses to ramp distension of the ex vivo colon and single unit analysis indicated that an impaired mechanosensitivity of low-threshold and wide dynamic range fibers may underlie the afferent hyposensitivity in the diabetic colon. Fewer PGP 9.5- or CGRP-immunoreactive fibers and lower protein level of PGP 9.5 were found in the colon of diabetic rats.

**Conclusions:**

These observations revealed the distinctive feature of colonic neuropathy in short-term diabetic rats that is characterized by a diminished sensory innervation and a blunted mechanosensitivity of the remnant sensory nerves.

## Background

Diabetic neuropathies are heterogeneous, affecting different parts of the peripheral nervous system and resulting in diverse symptoms. Most common among the neuropathies are chronic sensorimotor distal symmetric polyneuropathy and autonomic neuropathies. Somatic sensory neuropathy is known to result in neuropathic pain, loss of sensation and contribute to the development of diabetic foot ulceration. The manifestations of visceral neuropathy are often more unpredictable, but gastrointestinal (GI) symptoms such as gastroparesis, postprandial fullness, nausea, vomiting, bloating, diarrhea and constipation are amongst the most common clinical presentations of diabetes. The symptoms are often severe and substantially decrease the quality of life [1].

The mechanisms underlying the pathogenesis of the GI symptoms in the course of diabetes are undoubtedly multifactorial, including motor dysfunction, altered visceral sensitivity, altered gut hormone secretion, central neuroplastic changes as well as psychological and genetic factors (see references in [2]). As clinical management of these patients is often challenging, a better understanding of GI sensory processing in the diabetic condition is needed.

Alterations in upper GI sensation in diabetic patients have been studied vigorously. An increased sensory response to gastric distension was observed in diabetic patients [3, 4]. In contrast, hyposensitivity in the oesophagus and duodenum to various stimuli including mechanical, thermal and electrical stimulations was observed in diabetic patients with evident DAN and GI symptoms [5]. More recently, functional brain imaging studies have provided evidence that the CNS processing of visceral sensation is altered in diabetic patients with upper GI symptoms [6, 7].

Sensory deficits in the lower GI tract have also long been recognized and are suspected to be associated with symptoms such as diarrhea and constipation in diabetic patients [8–10]. However, few studies have directly assessed the morphological and functional changes in the primary afferent nerves innervating the lower GI tract and there is a lack of mechanistic understanding of the sensory deficits of the lower GI in diabetic conditions. Previously, Beyak and colleagues recorded the mechanosensitive response of rectal afferents in a flat-sheet rat rectum preparation and found that streptozotocin (STZ)-induced experimental diabetes selectively impaired the detection of low threshold ‘physiological’ rectal distention, such as that which might occur during rectal filling [11]. In contrast, Grabauskas et al. reported that a diminished KV4.2 current in DRG neurons might underlie colorectal hypersensitivity in STZ-induced diabetic rats [12]. In the present study we aim to explore visceral sensitivity and the underlying morphological and funtional alterations in the afferent innervation of the colorectum in the diabetic condition. To this end, we compared colorectal distension (CRD)-induced visceromotor response (VMR) in vivo, the mechanosensitivity of the colonic afferent nerves in the ex vivo colon preparations as well as the expression of protein gene product 9.5 (PGP 9.5) and calcitonin gene-related peptide (CGRP) in the colon of STZ-induced short-term diabetic and age-matched vehicle-treated rats.

## Results

**Fig. 1:**
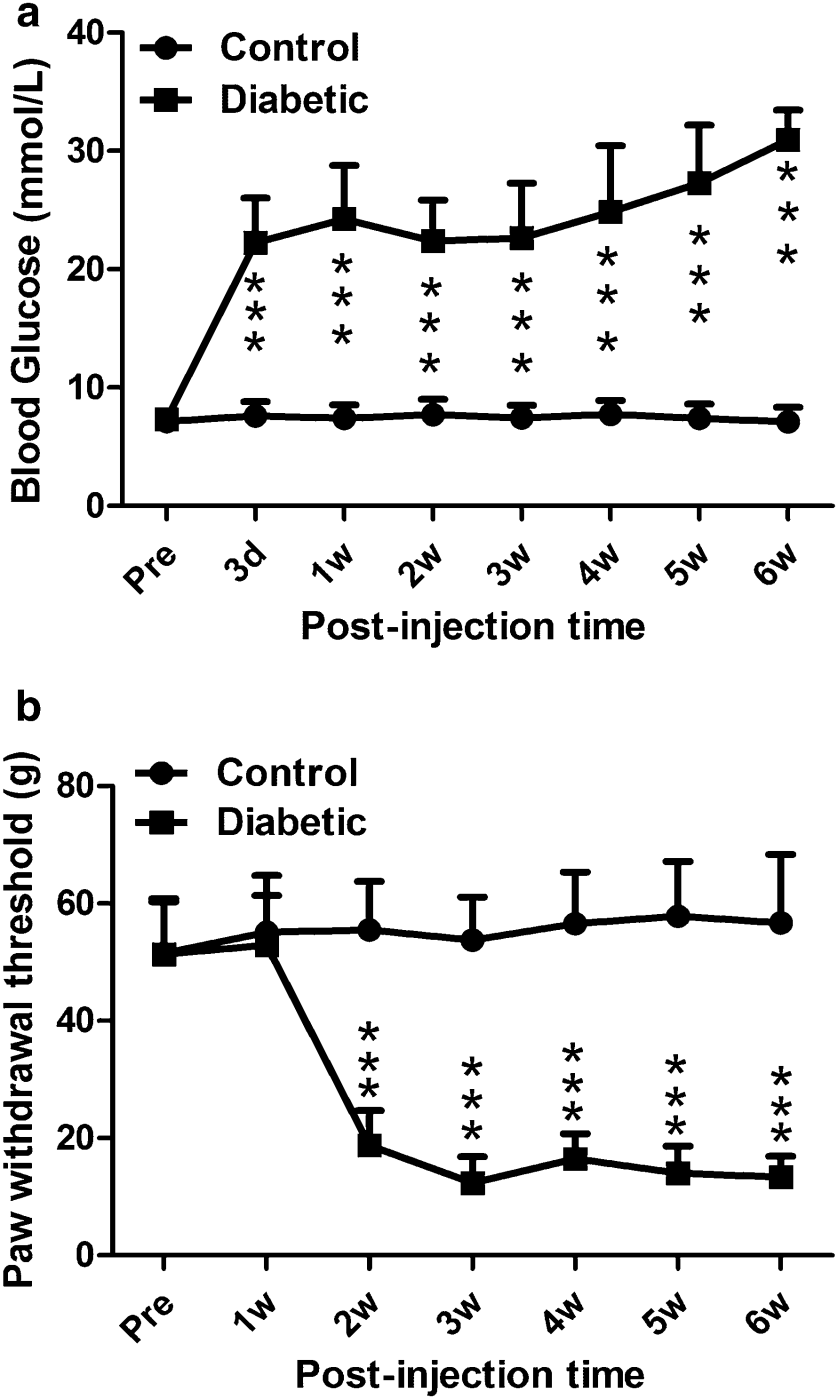
Effects of single STZ injection (50 mg/kg, i.p.) on rat blood glucose level and paw withdrawal threshold. **a** Blood glucose level was significantly increased since day 3 after STZ injection. **b** Paw withdrawal threshold was significantly decreased since the second week after STZ injection. ***P < 0.001, two-way ANOVA with Bonferroni’s posttest

About sixty-seven percent of rats (27/40) treated with STZ displayed sustained hyperglycemia (>15 mmol/L) since day 3, whereas the blood glucose level of the control rats remained unaltered (Fig. 1a). Concomitantly, the mean paw withdrawal threshold of the diabetic group was significantly lower than that of the control group (Fig. 1b). These data are indicative of the development of somatic mechanical hypersensitivity in STZ-induced short-term diabetic rats.

### Decreased VMR to CRD in diabetic rats

**Fig. 2:**
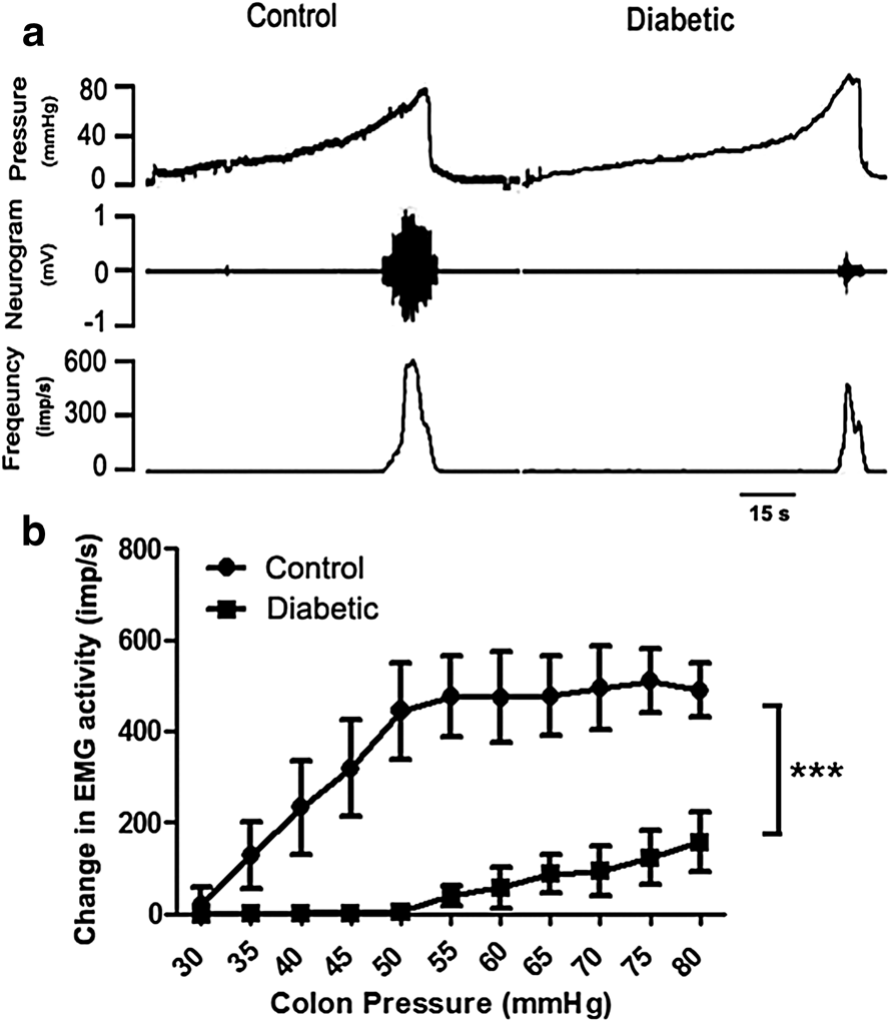
The visceromotor responses (VMR) to colorectal distension in diabetic and control rats. **a** Representative recordings of CRD-induced VMRs in control and diabetic rats. **b** The relationship between the VMR rate and the distention pressure in age-matched control (n = 7) and diabetic (n = 9) rats. A significant decrease in VMR occurred in diabetic rats compared with control rats. ***P < 0.001, un-paired t test of the area under curves (AUC)

Visceromotor responses to colorectal distension were examined in control and diabetic (3–6 weeks after injection of STZ) rats. As shown in Fig. 2a, the VMRs had a threshold of approximately 30.7 ± 3.6 mmHg and the amplitude increased linearly with the distension pressure in non-diabetic rats, while VMRs had an threshold of 50.4 ± 3.8 mmHg in diabetic rats which was significantly higher than that in the non-diabetic rats (P < 0.001, unpaired *t* test). Furthermore, the averaged pressure-response curve of VMRs was shifted downward in diabetic rats compared with the non-diabetic rats. Unpaired t-test of the area under the curves (AUC) indicated that the amplitude of VMR was significantly lower in diabetic than in control rats (P < 0.001, Fig. 2b). These results showed that the pseudoaffective responses to colorectal distension were diminished in early diabetes.

### Decreased mechanosensitivity of pelvic afferents innervating colon in diabetic rats

**Fig. 3:**
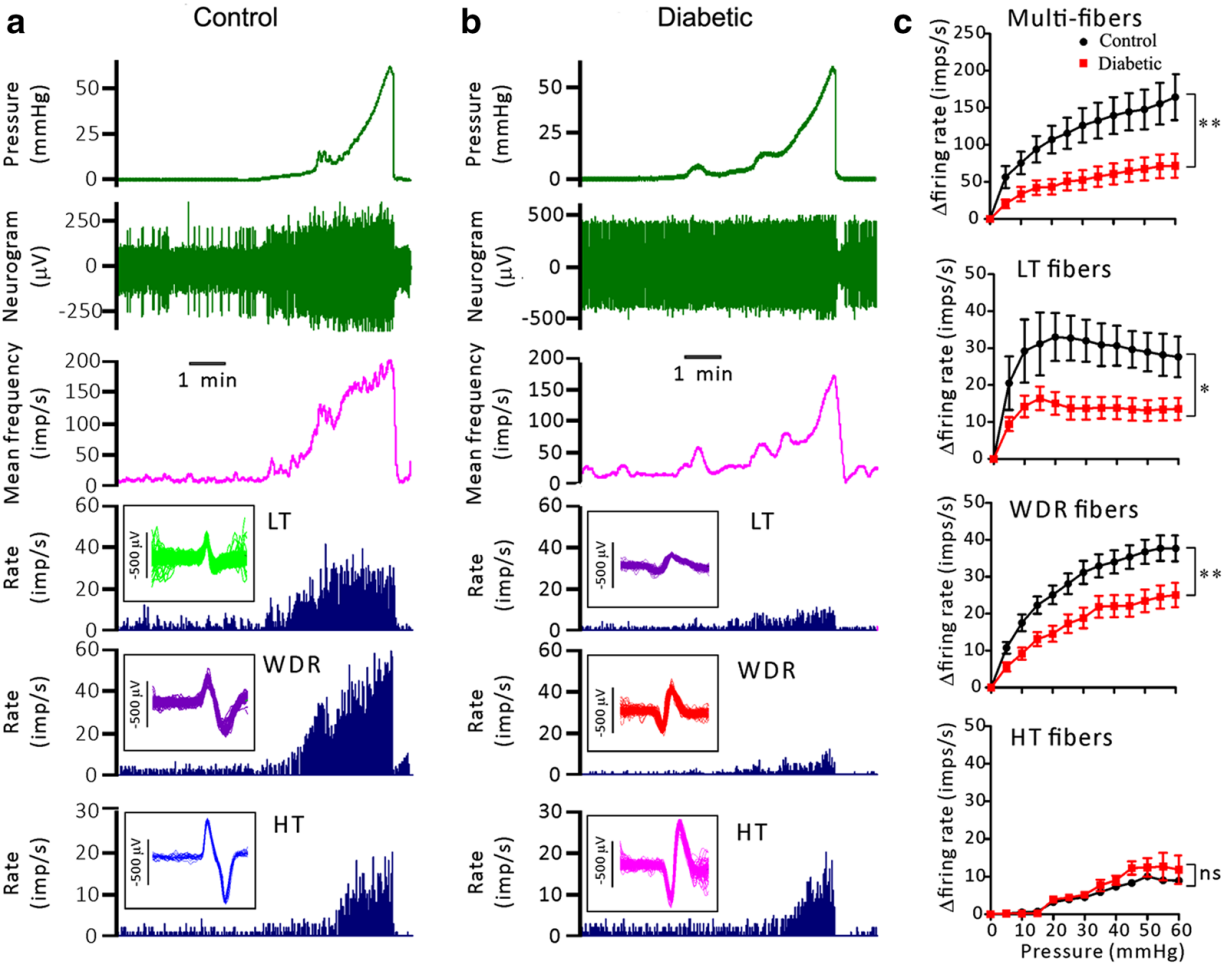
The mechanosensitivity of the pelvic afferent nerves in the ex vivo rat colorectum preparations. **a** and **b** show representative recording of multiunit afferent activity during ramp distension of the colorectum. From *top* to *bottom* are traces of the pressure, raw (multiunit) neurogram, the multiunit discharge frequency and the discharge rate of low threshold (LT), wide dynamic range(WDR) and high threshold(HT) units discriminated through principal component analysis. **c** shows the averaged pressure-response curves of multiunits (n = 17 and 23), LT (n = 6 and 9), WDR (n = 22 and 22) and HT units (n = 3 and 6) of the control and diabetic groups.*P < 0.05; **P < 0.01, un-paired t test of AUC

To test the possibility that impaired afferent innervation of the colorectum may underlie the diminished VMRs in diabetic rats, we compared the mechanosensitivity of pelvic afferents in the ex vivo colon preparations from diabetic and control rats. As shown in Fig. 3a, b, which are examples of multiunit activity in control and diabetic preparations, the colonic afferent nerves had a low level of irregular spontaneous activity (Control: 17 ± 8.9 imp/s, Diabetic: 23 ± 7.9 imp/s; P > 0.05, unpaired t test) and ramp distension of the colon induced an increase in the firing rate. The averaged pressure-response curve of the multiunit activity was shifted downward in the diabetic preparations compared to the control preparations. Unpaired t-test of AUC indicated that distension-induced multiunit activity was significantly lower in diabetic than in control preparations (P < 0.01, Fig. 3c).

Through the principal component analysis, two to four mechanosensitive units were identified in each multiunit recording. Colonic afferent fibers consist of three distinct populations according to the profiles of the responses to distension, namely low threshold (LT) fibers, wide dynamic range (WDR) fibers and high threshold (HT) fibers (Fig. 3a, b). Low threshold (LT) units were characterized by a very LT to distention, reaching a maximal firing rate at about 20 mmHg. There was no further increase in discharge in these afferents with further increases in pressure up to 60 mmHg. High threshold (HT) units had thresholds >20 mmHg, but the responses to encoded stimulus intensities were relative low. Wide dynamic range (WDR) units had a low threshold, however, continued to encode stimulus intensities across the range, and their pressure–response relationship mimics that of the whole-nerve activity.

Among the 31 single fibers in control rats, 6 (19.3 %) LT units, 22 (71.0 %) WDR units and 3 (9.7 %) HT units were identified. Similar proportions of LT (9/37, 24.3 %), WDR (22/37, 59.5 %) and HT (6/37, 16.2 %) units were identified in diabetic rats. There was no significant difference in the distribution of three types of response profile in diabetic and control rats (p = 0.629, Fisher’s exact test). However, the pressure-response curves of LT and WDR units of diabetic rats were shifted downward (Fig. 3c) and the corresponding AUC values were significantly smaller as compared to those of the control rats (P < 0.05 and P < 0.01 for LT and WDR units, diabetic vs control, unpaired t test, Fig. 3c). The pressure-response curves of the HT units of diabetic and non-diabetic rats were not significantly different. These results suggest that the mechanosensitivities of the LT and the WDR afferent fibers of the colon were blunted in the diabetic condition.

### Decreased PGP 9.5 and CGRP expression in the colon of diabetic rats

**Fig. 4:**
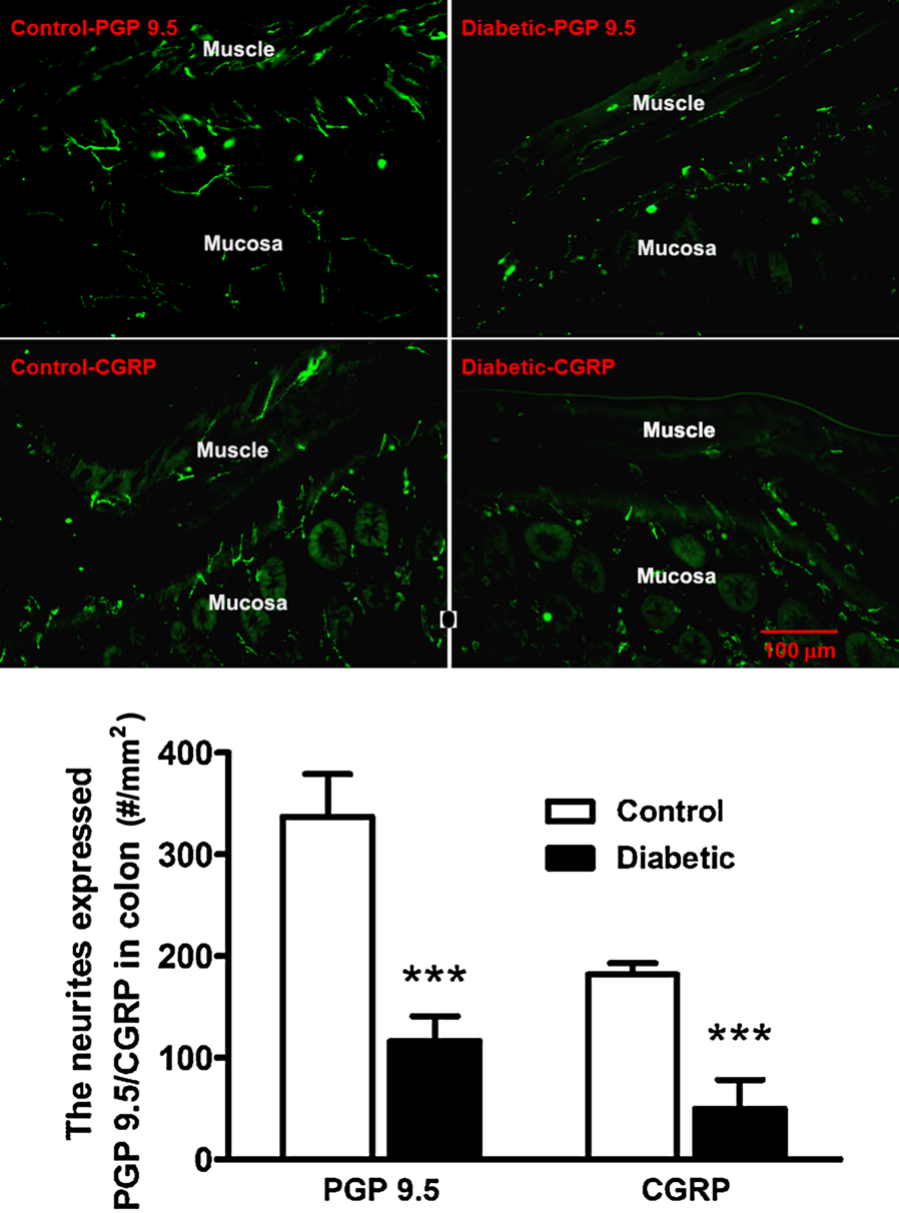
The PGP 9.5- or CGRP- immunoreactive neurites in the colon from control and diabetic rats. *Top* Representative microphotographs of PGP 9.5- or CGRP-immunoreactive neurites in the colon from control and diabetic rats. *Bottom* The *bar graph* showing the number of neurites expressed PGP 9.5 or CGRP in control and diabetic rats. Both PGP 9.5- and CGRP-immunoreactive neurites were much more less in all layers of the colon in diabetic rats (n = 7) than those in control rats (n = 5). ***p < 0.001 vs. control, unpaired t test

**Fig. 5:**
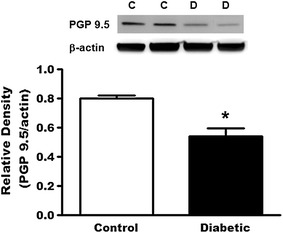
The protein levels of PGP 9.5 in the colon from control and diabetic rats. *Top* Representative bands of PGP 9.5 (MW: 27 kDa) and β-actin (MW: 42 kDa) in the colon from control and diabetic rats. *Bottom Bar graph* showed the relative density of PGP 9.5 in the colon from control and diabetic rats. Protein level of PGP 9.5 was decreased in the colon from diabetic rats (n = 6) compared to control rats (n = 4). *P < 0.05 vs. control, unpaired t test. *C* control, *D* diabetic

To investigate the possibility that the density of afferent innervation of the colon might be altered in the diabetic condition, we performed immunofluorescent staining for PGP 9.5 (a general neuronal marker) and CGRP (a C- and Aδ sensory fiber marker) to visualize afferent neurites within the colon tissue. Neurites immunoreactive for PGP 9.5 or CGRP could be detected in the mucosa, the submucosa and the muscle layer of the colon and appeared to be more sparse in the diabetic than in the control rat colon tissue (Fig. 4 top). Neurite counting showed a significant reduction of neurite density in the diabetic (116.5 ± 9.9 PGP 9.5^+^-neurites/mm^2^ and 49.7 ± 10.9 CGRP^+^-neurites/mm^2^) as compared to the control colon tissue (336.8 ± 20.9 PGP 9.5^+^-neurites/mm^2^ and 182.0 ± 5.6 CGRP^+^-neurites/mm^2^, P < 0.001, unpaired t test, Fig. 4 bottom). In addition, western blot analysis revealed a significant reduction in PGP 9.5 expression in the diabetic rat colon (Fig. 5).

## Discussion

Somatic neuropathy induced by diabetes has been studied extensively in recent years [13–18]. In contrast, diabetic neuropathy in visceral organs such as the gastrointestinal (GI) tract is still poorly understood. In the present study, we found that in the early stage (3–6 weeks) of STZ-induced diabetes, rats developed colorectal hyposensitivity with concomitant decreases of the mechanosensitivity and the density of primary afferent fibers innervating the colorectum.

In rodents, the most common experimental model of short-term insulin-deficient diabetes is the elimination of pancreatic β islet cells by administration of STZ [19]. In the present study, rats treated with STZ (50 mg/Kg, i.p.) developed sustained hyperglycemia since day 3 and in keeping with previous reports they developed somatic mechanical hypersensitivity [20].

We assessed the sensitivity of the lower GI tract in the diabetic rats by recording the viscero-motor responses (VMR, i.e., abdominal EMG) during gradual colorectal distension. CRD-induced VMR, akin to the paw withdrawal reflex upon noxious pinch, is a nocifensive reflex elicited by nociceptive signals originating from the colorectum. We found that compared to the non-diabetic rats, diabetic rats showed a relative insensitivity to CRD, as manifested by a higher distension pressure required to evoke VMR (higher threshold) and a lower VMR magnitude. Notably, such colorectal hyposensitivity occurred at 3–6 weeks following STZ treatment, which was considered as the early stage of STZ-induced diabetes [11, 16]. In contrast, Grabauskas et al. [12] and Romanovsky et al. [21] reported colorectal hypersensitivity in rats 8 and 2 weeks after STZ injection, respectively. The reasons for these discrepancies remains to determined, however, it might be due to the different pattern of stimulation (i.e., continuously gradual distention of colon in current study vs. intermittently graded distention of colon in other studies). Another possibility is that colorectal sensitivity may alter dynamically as diabetes progresses because the experiments tested at different time points after STZ injection were seen in these studies. In this regard, it is interesting to note that in previous reports somatic thermal hyperalgesia [13, 18] and hypoalgesia [13, 22] were described in short-term (2–8 weeks) and longer-term (≥12 weeks) STZ-induced diabetic rats, respectively.

VMR is a nocifensive reflex that involves both peripheral and central neuronal pathways. To address the contribution of the peripheral nerves in the colorectal hyposensitivity in short-term diabetes, we examined the distension-induced pelvic nerve discharge in the ex vivo colorectum preparation taken from control and diabetic rats. Multiunit recording demonstrated a significantly diminished afferent nerve response to ramp distension of the colorectum. Single-unit analysis revealed a lower mechaosensitivity of the low threshold (LT) and the wide dynamic range (WDR) fibers in diabetic rats than in non-diabetic rats. These results are consistent with a recent study by Beyak et al., who studied the stretch responses of pelvic afferents in a flat-sheet rat rectum preparation and found that the LT and the WDR fibers of diabetic rats (4–8 weeks after STZ injection) were less sensitive to stretch than those of non-diabetic rats [11]. These findings indicate that functional deficits of the primary afferents contribute to the colorectal hyposensitivity seen in the short-term diabetic condition.

Previous studies showed that loss of intraepidermal afferent fibers led to somatic mechanical hypoalgesia in STZ-induced diabetic rats (12 weeks after STZ injection) and mice (4–8 weeks after STZ injection) [22, 23]. To explore the possibility that loss of afferent fibers may also underlie the colorectal hyposensitivity in the early diabetic rats, we conducted immunofluorescent staining of nerve fibers within the colorectum using PGP 9.5 as a pan-neuronal marker and CGRP as a selective marker for Aδ- and C-fibers [24]. We found that the density of CGRP-immunoreactive neurites in the colon tissue was significantly decreased in the diabetic than in the non-diabetic rats, as was the density of PGP 9.5-immunoreactive neurites. In addition, western blot analysis showed that the protein level of PGP 9.5 was decreased in the colon from diabetic rats. These findings confirm a loss of peripheral nerve fibers, especially the nociceptive sensory fibers of the colorectum in early diabetic rats.

Intriguingly, we found that the rats early in the course of diabetes showed somatic mechanical hypersensitivity but visceral mechanical hyposensitivity. This indicates that the different responses of nervous systems to hyperglycemia do occur between somata and viscera. One possibility is the different characteristics of primary afferent nerves between somata and viscera. Most of the sensory nerves innervated to viscera are c-(unmyelinated) and Aδ (thin myelinated) fibers, while the sensory nerves innervated to somata are thick myelinated Aβ fibers that usually contribute to the somatic inflammatory and neuropathic pain except for the c- and Aδ fibers. Another possibility is the different changes in microenvironments surround the afferents between samata and viscera after diabetes. The mechanisms underlying the different responses will be further explored in the future studies.

Painful or painless peripheral neuropathy is a frequent and severe complication of diabetes. Although chronic hyperglycemia play an important role in the pathogenesis of diabetic neuropathy, other factors, such as insulinopenia, dyslipidemia, insulin resistance, oxidative stress and abnormalities in insulin-like growth factors production, are likely to play a precipitating role. The mechanisms underlying visceral diabetic neuropathy requires large longitudinal studies to be explored.

## Conclusion

The present study provides data that shows diminished visceromotor responses to colorectal distension in diabetic rats 3–6 weeks after STZ injection. Concomitantly, the mechanosensitive responses of the low threshold and the wide dynamic range afferent fibers of the colorectum were significantly decreased, and the density of PGP 9.5- or CGRP-immunoreactive fibers was diminished in the diabetic rats. These findings suggest that visceral hyposensitivity may develop early in the course of diabetes due to both functional and structural deficits of the primary afferent nerves.

## Methods

Male Sprague–Dawley rats (190–210 g) were housed (three animals per cage) in a temperature controlled room (22–25 °C) illuminated from 07:00 to 19:00 h. Food and water were available ad libitum. All animal care and experimental protocols complied with the Guiding Principles in the Care and Use of Animals and the Animal Management Rule of the Ministry of Public Health, People’s Republic of China (documentation 545, 2001).

### Induction of diabetes

The most commonly used experimental model of diabetes in rodents is the elimination of pancreatic β islet cells by administration of STZ. STZ-induced diabetes produces early functional and biochemical abnormalities within peripheral nerves of rats and mice similar to those observed in human diabetic neuropathy [19]. Diabetes was induced in male Sprague–Dawley rats by an intraperitoneal (i.p.) injection of STZ (50 mg/kg, n = 40) dissolved in 0.01 M cold citrate buffer (pH 4.0). Control rats (n = 20) were injected with the same volume of vehicle (0.01 mol/L citrated buffer). From day 3 after injection, blood glucose level was checked weekly using the glucose-oxidase test strip and a reflectance meter (Roche Diagnostics GmbH, Ireland) on blood samples obtained from a tail prick. The animals showing a sustained hyperglycemia (27/40, 67.5 %) were used for comparison of the visceral sensitivity and the expression of neuronal markers 3–6 weeks after injection of STZ or vehicle.

### Colorectal distention (CRD)-induced visceromotor responses (VMR)

CRD-induced VMR is a pseudoaffective response induced by noxious stimulation of the viscera and has been used extensively to evaluate visceral sensitivity in experimental animals [25]. To assess the changes in colorectal sensitivity in early diabetes, STZ-treated rats with sustained hyperglycemia (n = 9) were anesthetized by i.p. injection of pentobarbital sodium (60 mg/kg) and the rats’ body temperature were kept around 37.5 °C using a thermostatic blanket. A lubricated polyethylene balloon (3 cm long, 1.5 cm max diameter) was inserted into the colorectum (the distal end of the balloon was about 2 cm proximal to the anus) for gradual CRD. The balloon was connected to a pressure control device and a pressure sensor. Two silver wire electrodes were implanted into the musculus obliques externus abdominis and rectus abdominis and a reference electrode was inserted subcutaneously to record the eletromyogram (EMG) induced by CRD. The balloon pressure and EMG signals were fed via the Powerlab interface into a computer and analyzed by Chart software (Powerlab; AD Instrument, Bella Vista, Australia). Following the surgical preparation which is usually lasted about 1 h, the rat was kept under light anesthesia via inhalation of 1 % isoflurane and was allowed at least 30 min to acclimatize before the test began. CRD was performed via gradually inflating the balloon up to a pressure of 80 mmHg within 2 min and this was repeated at 10 min intervals. Generally, 6 trials were run to achieve a stable response (less than 20 % variability between the last two trials). The same experimental paradigm was completed with age-matched control rats (n = 7).

### The ex vivo colon preparation and pelvic afferent nerve recording

At 4–5 weeks after induction of diabetes mellitus, rats (n = 5) were deeply anaesthetized with pentobarbital (80 mg/kg, i.p.). The abdominal cavity was exposed by a midline incision on the abdominal wall. The colon 3 cm proximal to the anal sphincter with the attached pelvic ganglia and pelvic nerves was removed and was placed into a recording chamber (20 mL), where it was superfused continuously at a rate of 15 mL/min with oxygenated Krebs solution (composition in mmol/L: NaCl 113, KCl 5.9, CaCl_2_ 1.25, MgSO_4_ 1.2, NaH_2_PO_4_ 1.2, NaHCO_3_ 25, Glucose 11.5). Chamber temperature was kept at 34 °C. A fine nerve branch emanating from the pelvic ganglion was dissected and recorded using a glass suction electrode filled with the buffer. The colon was catheterized at the both ends in order to apply repeated mechanical stimulation by ramp distension (0–60 mmHg) at an interval of 15 min. Neuronal signal was amplified (Neurolog NL102, Digitimer, UK), filtered (band pass 300–3000 Hz) and fed into a computer via Micro1401 interface (Cambridge Electronic Design, UK). Afferent discharge was saved and processed using spike 2 software (version 5.14, Cambridge Electronic Design, UK). The activity of single units in each recording was discriminated by performing the principal component analysis of the contour of individual spikes as described previously [26]. The same experimental paradigm was completed with vehicle-treated control rats (n = 4).

### Immunofluorescence (IF) staining

Immunofluorescent staining of the general neuronal maker PGP 9.5 and the specific sensory marker CGRP for C- and Aδ-fibers [24] were performed in colon from diabetic rats (4–5 weeks after induction of diabetes, n = 7) and age-matched control rats (n = 5). Under anesthesia with pentobarbital, rats were transcardially perfused with saline followed by 4 % paraformaldehyde and 0.14 % picric acid in phosphate buffer (PB, 0.1 mol/L, pH 7.4). The colon were removed and post-fixed in the same fixative overnight at 4 °C and then cryoprotected with 30 % sucrose in 0.1 mol/L PB overnight at 4 °C. The samples were cut at 16 μm for staining as described previously [27]. The sections were first incubated with 0.05 mol/L phosphate-buffered saline (PBS) containing 10 % normal goat serum and 0.5 % TritonX-100 at room temperature for 1 h to block non-specific binding and this was followed by incubation with primary rabbit anti-PGP 9.5 antibody (1:1000, abcam, Cambridge, MA, USA) or rabbit anti-CGRP antibody (1:500, abcam, Cambridge, MA, USA) at 4 °C overnight. The sections were rinsed with PBS for four times and were then incubated with goat anti-rabbit Alexa fluor 488 secondary antibody (1:1000; Molecular Probes-Invitrogen, Eugene, OR, USA) at room temperature for 1 h. After washing with PBS the sections were mounted on glass slides and viewed under the fluorescent microscope (Leica DM2500, Leica Microsystems Limited, USA) and the digital images were analyzed using Leica application suite version 4.3 (Leica Microsystems Limited, USA). Nerve fiber density was evaluated by counting the number of neurites expressed PGP 9.5 or CGRP. The number of neurites/mm^2^ was calculated and averaged from six randomized fields for each rat. Neurites appeared as thin, relatively straight green lines, distinct from the background, sometimes beaded with varying width, and sometimes branched. Each straight continuous or beaded line was counted as one neurite, while the tip of each branch was counted as another neurite, described as previous study [27]. Counts were done by two investigators who blinded to the group assignment and calculations were therefore done using averages of the two sets of counts.

### Western blot

Diabetic (n = 6) and control (n = 4) rats were deeply anesthetized and their colons were removed, frozen immediately on dry ice and stored at −80 °C. The samples were homogenized in lysis buffer containing 20 mmol/L Tris–HCl (pH 8.0), 150 mmol/L NaCl, 1 mmol/L EDTA, 1 % NP-40, 1 mmol/L PMSF, protease inhibitor cocktail (Sigma, St. Louis, MO, USA) and phosphatase inhibitor cocktail (Thermo scientific, Indianapolis, IN, USA) for 1 h at 4 °C. The lysates were centrifuged at 10,000*g* for 30 min at 4 °C and the concentration of protein in each supernatant was determined using a BCA assay (Pierce, Rackford, IL, USA). Thirty-microgram aliquots were separated on 4–20 % Tris–glycine ready gels (Bio-rad, Hercules, CA, USA), the separated proteins were transferred from the gel to the surface of Nitrocellulose membranes (Bio-rad). The membranes were blocked with 5 % fat-free dry milk in Tris-buffered saline (TBS) containing 0.1 % Tween-20 for 1 h, and were then incubated for 48 h at 4 °C with primary rabbit anti-PGP 9.5 antibody (1:200, abcam, Cambridge, MA, USA) and with mouse anti-β-actin antibody (1:2000, abcam) for 2 h at RT, respectively. Bound primary antibodies were detected with HRP-conjugated anti-rabbit or anti-mouse secondary antibody (1:5000, Bio-rad). Immunoreactive bands were visualized using enhanced chemiluminescence (Thermo scientific) and digital imaging was captured with Image Quant LAS 4000 mini (GE Healthcare, Life Science, USA). The density of specific bands was measured with NIH ImageJ (http://rsb.info.nih.gov/ij/) software and was normalized against a loading control (β-actin). We tried but failed to get the CGRP signal (band not at theoretic molecular weight, data not shown).

### Paw pressure test

One day before VMR measurement, mechanical paw withdrawal thresholds (PWTs) were determined by probing the left hind paw with calibrated Von Frey hairs as described previously [28] in diabetic (n = 9) and control (n = 7) rats (same rats for VMR study). Von Frey hairs of increasing size (hence of increasing force) were applied to the plantar surface of the left hind paw until the animal withdrew the paw. The smallest size of the von Frey hairs required to produce a withdrawal reflex was recorded as the threshold.

### Statistical analysis

Data were presented as mean ± SD. Statistical analysis of the data sets was carried out using Graphpad Prism version 3.0 (Graphpad software, La Jolla, CA, USA). Two-way ANOVA with Bonferroni’s posttest was used to assess the difference between experimental groups for blood glucose level and paw withdrawal threshold. To analyze VMR and pelvic nerve responses, an X–Y plot analysis was first conducted to measure the increases in afferent discharge rate from the baseline as the intraluminal pressure rose by every 1 mmHg. Such analysis gave rise to individual pressure-afferent response curves and then the group (average) pressure-afferent response curves. For area under the curve (AUC) analysis, the AUC of individual pressure-afferent response curve was determined and was then averaged. Unpaired t-test was used to assess the difference in averaged AUC between experimental groups. For PGP 9.5/CGRP expression analysis, unpaired t-test was used to assess difference between experimental groups. Significance was set at p < 0.05.
